# Whole Genome Analysis of *Pediococcus acidilactici* XJ-24 and Its Role in Preventing *Listeria monocytogenes* ATCC^®^ 19115^TM^ Infection in C57BL/6 Mice

**DOI:** 10.3390/antibiotics14030323

**Published:** 2025-03-19

**Authors:** Weizhong Hu, Shuxin Zhou, Amel Ibrahim, Guannan Li, Sameh Awad, José Ramos-Vivas, Jianquan Kan, Muying Du

**Affiliations:** 1 College of Food Science, Southwest University, Chongqing 400715, China; weizhong_hu1030@163.com (W.H.); zsx18200391607@email.swu.edu.cn (S.Z.); kanjianquan@163.com (J.K.); 2 Chinese-Hungarian Cooperative Research Center for Food Science, Southwest University, Chongqing 400715, China; 3 Chongqing Key Laboratory of Speciality Food Co-Built by Sichuan and Chongqing, Chongqing 400715, China; 4 Faculty of Agriculture, Alexandria University, Alexandria 21500, Egypt; amal.ibrahim@alexu.edu.eg (A.I.); sameh.awad@alexu.edu.eg (S.A.); 5 College of Sericulture, Textile and Biomass, Southwest University, Chongqing 400716, China; gn9899@163.com; 6 Research Group on Foods, Nutritional Biochemistry and Health, Universidad Europea del Atlántico, 39011 Santander, Spain; jose.ramos@uneatlantico.es

**Keywords:** probiotic, whole-genome analysis, intestinal barrier, intestinal microbiome

## Abstract

**Background/Objectives:** As probiotics gain prominence in the prevention and treatment of intestinal diseases, their protective effects against pathogens and influence on host health have drawn significant attention. This study investigates the genomic characteristics and functional potential of *Pediococcus acidilactici* XJ-24 (XJ-24) in the prevention of *Listeria monocytogenes* (LM) infection in mice. **Methods/Results:** Whole-genome analysis confirmed the safety and probiotic properties of XJ-24, including acid and bile salt tolerance, antimicrobial activity, and safety. In vivo, C57BL/6 mice challenges indicated that XJ-24 significantly reduced LM colonization, suppressed pro-inflammatory cytokines (IL-1β, IL-6, TNF-α, IFN-γ), alleviated colon and spleen tissue damage, and maintained intestinal barrier integrity by upregulating tight junction proteins (Occludin, Claudin-1, ZO-1). Moreover, XJ-24 modulated gut microbiota composition by increasing beneficial taxa while reducing harmful bacteria. Correlation analysis highlighted a positive association between *Lachnospiraceae* and tight junction proteins. **Conclusions:** These findings demonstrate the potential of XJ-24 as a functional probiotic for preventing LM infection and provide a basis for further clinical exploration.

## 1. Introduction

*Listeria monocytogenes* is a Gram-positive foodborne pathogen transmitted through contaminated food, posing significant risks to immunocompromised individuals, pregnant women, the elderly, and neonates [1]. Upon ingestion, *L. monocytogenes* colonizes the gastrointestinal tract, disrupting the intestinal barrier by inducing an inflammatory response, increasing intestinal permeability [2]. This allows pathogens, toxins, and other harmful substances to invade host tissues, organs, and the bloodstream, ultimately leading to systemic dysfunction [2,3,4]. The high mortality rate of listeriosis and its frequent association with global food contamination underscores the significance of *L. monocytogenes* as a critical food safety and public health issue [5]. Despite these concerns, there is currently no clinically approved vaccine for listeriosis, forcing severe cases to become dependent on a combination of antibiotic therapies [6]. Furthermore, the growing challenge of antibiotic resistance highlights the urgent need for innovative, safe, cost-effective strategies to prevent and treat listeriosis [7].

Probiotics are live commensal bacteria that defend against gastrointestinal infections and offer health benefits when consumed in adequate amounts [8]. Recently, probiotics have garnered significant attention as natural and safe biological agents for preventing *L. monocytogenes* infections [9,10,11]. For instance, *Lactobacillus delbrueckii* UFV-H2b20, when administered orally, enhances immune functions and protects germ-free mice from infections caused by *L. monocytogenes* [12]; oral administration of *Akkermansia muciniphila* mitigated *L. monocytogenes* infection by strengthening intestinal barrier function and elevating linoleic acid levels in the gut [7]. Furthermore, the Becattini study highlighted the importance of gut microbiota in defending against *L. monocytogenes* infection [13]. Li reported that oral intake of *Lactiplantibacillus plantarum* modulated the gut microbial community in mice, promoting beneficial bacteria while suppressing harmful bacteria to counteract *L. monocytogenes* infections [2]. A key infection mechanism of *L. monocytogenes* involves breaching host barriers, such as the intestinal epithelium, blood-brain barrier, and placenta [2]. Probiotic supplementation provides multifaceted defenses, including competing with pathogens for adhesion sites, rebalancing gut microbiota, strengthening intestinal barrier function, and boosting mucosal immunity, thereby mitigating infection and associated pathologies [14,15,16,17]. In conclusion, probiotics hold significant potential for preventing and mitigating *L. monocytogenes* infections, establishing a valuable foundation for developing innovative biotherapeutic approaches.

Probiotic lactic acid bacteria have been widely documented for their efficacy in treating gastrointestinal disorders, with no reported adverse effects [18]. *Pediococcus acidilactici* is a lactic acid bacterium identified in numerous studies as a promising probiotic, exhibiting diverse beneficial functions [19,20,21]. For instance, *P. acidilactici* has the potential to prevent hyperglycemia, hypercholesterolemia, and gastrointestinal infections [18]. *P. acidilactici* MRS-7 has been demonstrated to enhance tight junction protein levels and influence gut microbiota composition, thereby mitigating rod trichothecene-induced jejunal damage in mice [22]. On the other hand, *P. acidilactici* BA28 has effectively eradicated *Helicobacter pylori* infections and reversed peptic ulcer disease [23]. Despite the numerous functional properties attributed to *P. acidilactici*, its potential role in preventing *L. monocytogenes* infections remains largely unexplored, highlighting the need for further research.

Furthermore, evaluating the strain’s safety and probiotic characteristics is essential to confirm its suitability for application in humans and animals [24]. Currently, whole-genome analysis is considered the gold standard for assessing the safety of probiotic strains [25]. This approach is efficient and cost-effective, enabling the rapid screening of strains by analyzing probiotic molecular mechanisms through genome sequencing and functional annotation [26]. An important focus of bacterial genome research is the discovery of molecular markers linked to probiotic characteristics, including acid and bile tolerance, antimicrobial production, and safety [27]. Additionally, it is crucial to identify any potential antibiotic-resistance genes to ensure the strain’s safety and prevent the spread of antimicrobial resistance. Consequently, whole-genome analysis facilitates a comprehensive evaluation of probiotic safety and functional attributes, offering a scientific basis for selecting appropriate probiotic strains.

In our previous study, the strain *P. acidilactici* XJ-24 demonstrated strong in vitro inhibitory activity against *L. monocytogenes* and exhibited promising probiotic properties [28]. However, the lack of complete whole-genome information for this strain and the unexplored in vivo preventive effects against *L. monocytogenes* infections hinder any further applications for it. This study aims to thoroughly analyze the genome of *P. acidilactici* XJ-24 using whole-genome sequencing, identify key functional genes, and explore its in vivo mechanisms and effectiveness in preventing *L. monocytogenes* infection.

## 2. Results and Discussion

### 2.1. Genomic Features of XJ-24

**Figure 1:**
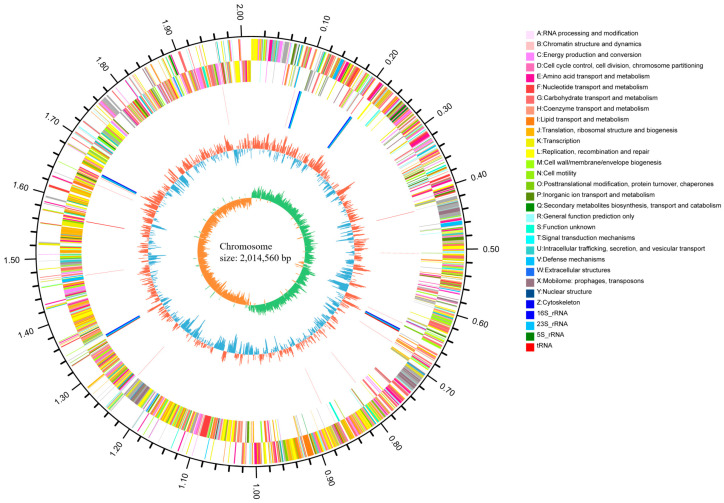
A circular genomic map of the XJ-24 chromosome.

The de novo assembly of PacBio sequencing reads for XJ-24 yielded a 2,014,560 bp circular chromosome with a GC content of 42.33% (Figure 1) and a 55,336 bp circular plasmid with a GC content of 40.36% (Figure S1). The genome consists of 2010 coding genes with an average length of 886 bp, including 15 rRNA genes (5 for 5S rRNA, 5 for 16S rRNA, and 5 for 23S rRNA), 58 tRNA genes, and 27 sRNA genes (Table 1). Functional annotation of the XJ-24 genome was conducted using the COG, KEGG, and GO databases. COG functional annotation showed that 1620 of the 2010 protein-coding genes (80.60%) were assigned to 23 subclasses within four major categories: metabolism (40.08%), cellular component (22.97%), information processing (25.31%), and others (11.65%) (Figure S2). GO functional annotation revealed 2144 genes associated with molecular function, 1079 genes linked to biological processes, and 797 genes related to cellular components (Figure S3). Note that the total number of genes exceeds the 2010 CDS genes identified initially because a single gene can be annotated to multiple GO categories, and multiple genes can share the same GO term. In the KEGG database, 1667 genes were annotated, among which 1150 genes (68.99%) were enriched in metabolic pathways (Figure S4). The complete genome sequence of XJ-24 has been submitted to the NCBI GenBank (Accession numbers: CP172259–CP172260).

### 2.2. Evaluation of Adaptability and Stress Response Genes in Human Gastrointestinal Environment

Probiotics are live microorganisms that confer health benefits to the host when consumed in adequate amounts [29]. Probiotics must possess several essential traits to exert their beneficial effects, including the capacity to tolerate the human gastrointestinal environment, safety, and functionality [24,30]. Bacteria exposure to acidic, low-temperature, or high-temperature conditions can damage the integrity and functionality of nucleic acids, lipids, and proteins, ultimately impacting bacterial viability [26]. However, bacteria can produce stress-response proteins to mitigate damage caused by environmental factors like pH and temperature fluctuations [24]. The genome of XJ-24 contains numerous genes that code for adaptive stress-response proteins, including six general stress response proteins, clp proteins, hslV and hslU proteins, various heat shock proteins (groES, groL, dnaK, dnaJ, hrcA), and cold shock proteins (cspA) (Table 2) [26,31]. Furthermore, research indicates that *P. acidilactici* possesses genes linked to stress resistance and mitigation [18,32]. Additionally, lactic acid bacteria possess various acid-resistance mechanisms, such as F_1_F_0_-ATPase and ornithine decarboxylase, to sustain a stable intracellular pH [24]. The genome of XJ-24 comprises a complete F_1_F_0_-ATPase proton pump system (atpA-atpH) and a Na^+^/H^+^ antiporter gene. These proteins play a critical role in responding to acidic stress by expending cellular ATP to extrude H^+^ from the cell, stabilizing intracellular pH [33]. In addition, the genome of XJ-24 harbors genes encoding the arginine/ornithine antiporter (arcD), glucosamine-6-phosphate deaminase (nagB), and CTP synthase (pyrG), which facilitate its resistance to bile salts in the host gastrointestinal tract [26]. Notably, bile salt hydrolase genes were not identified in the genome of XJ-24. This is consistent with previous findings that BSHs are also absent in other *P. acidilactici* strains, suggesting that this species may rely on alternative mechanisms, such as the aforementioned genes, to survive in bile-rich environments [18]. Previous in vitro evaluations confirmed that XJ-24 survives in low pH (2.5) and high bile salt (0.3%) environments [28]. These results indicate that XJ-24 possesses probiotic potential and can survive the digestive processes within the host.

### 2.3. Safety Assessment of XJ-24

Using the Resistance Gene Identifier (RGI) in the CARD, three antibiotic resistance genes—*sdrM*, *vanT* (from the *vanG* cluster), and *qacG*—were identified on the chromosome of XJ-24, with relatively low similarity ranging from 30% to 50% (Table S2). Importantly, these three antibiotic-resistance genes were neither located on the plasmid nor within genomic islands, insertion sequences (IS units), and transposons. Since horizontal transfer of antibiotic resistance is primarily associated with mobile genetic elements, particularly plasmid-mediated conjugation [34], the risk of horizontal transfer in XJ-24 is considered low. Furthermore, prior antimicrobial susceptibility testing (AST) demonstrated that XJ-24 is susceptible to various antibiotics, including penicillin, ceftriaxone, erythromycin, and chloramphenicol [28]. However, this study did not conduct AST specifically targeting the identified resistance genes, representing a limitation that warrants further investigation. Nevertheless, the observed antibiotic susceptibility suggests that these resistance genes may not confer significant resistance under the tested conditions.

Furthermore, based on the VFDB, 175 potential virulence factor genes were identified in the XJ-24 genome, each showing less than 75% similarity (Table S3). Many genes annotated as virulence factors were also classified in other functional databases, such as the COG database. According to COG database annotations, these genes are involved in cellular processes and signaling, information storage and processing, or metabolism, including posttranslational modification, protein turnover, chaperones (O), cell wall/membrane/envelope biogenesis (M), translation, ribosomal structure and biogenesis (J), carbohydrate transport and metabolism (G), lipid transport and metabolism (I), and transcription (K). These results on antimicrobial resistance, coupled with the absence of classical virulence factors, suggest that the probiotic strain XJ-24 has a favorable safety profile. However, the presence of unrecognized virulence factors cannot be entirely ruled out.

### 2.4. Bacteriocin of XJ-24

Antimicrobial activity is a key criterion in the selection of probiotics [35]. Previous research has demonstrated that the cell-free supernatant of XJ-24 exhibits potent antibacterial activity against *L. monocytogenes* (LM) [28]. Additionally, co-culture experiments in pasteurized milk revealed that XJ-24 exerts significant in situ inhibitory effects against LM [28]. Genomic analysis with the BAGEL4 platform further revealed the presence of a gene encoding Enterolysin A in the XJ-24 genome, which shares 96.08% protein identity with the published Enterolysin A sequence from *P. acidilactici* NGRI 0510Q (GenBank accession number: GAC46137.1) (Figure S2). Enterolysin A is a bacteriocin that can degrade bacterial cell walls and is commonly present in various bacterial genera, including *Enterococcus*, *Lactobacillus*, and *Pediococcus* [36,37]. Notably, Enterolysin A has been shown to effectively inhibit LM growth [38,39,40].

These findings underscore the potential of XJ-24 as a probiotic strain and its ability to inhibit LM.

### 2.5. Effects of XJ-24 Pretreatment on Physiological Indices in Mice Infected with LM

**Figure 2:**
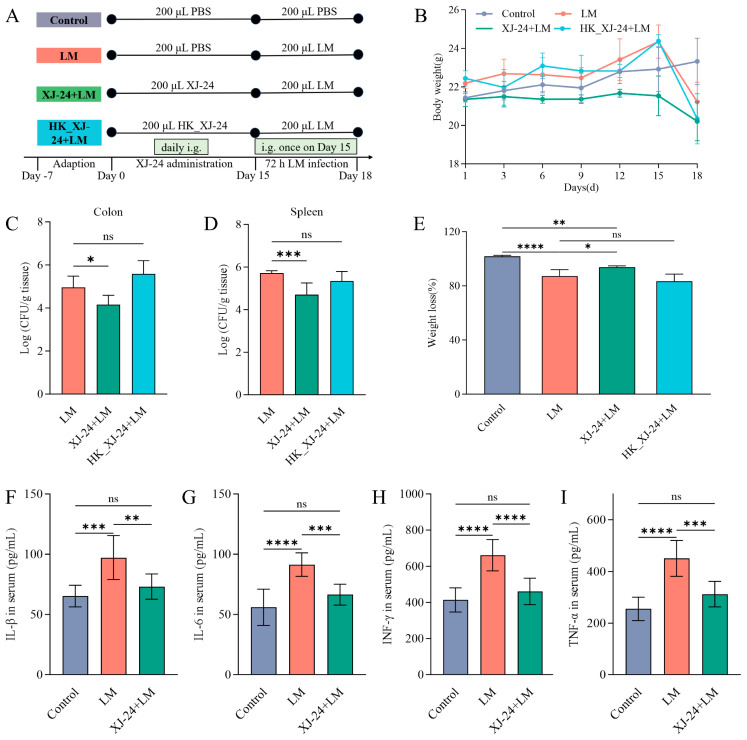
Body profiles of the mice: (**A**) The study design for the animal experiments, (**B**) Changes of body weight in each group, (**C**,**D**) LM translocation in the colon, spleen, and brain, (**E**) Weight loss was calculated as (weight on day 18/weight on day 15) × 100%, (**F**–**I**) Levels of inflammatory cytokines (IL-1β, IL-6, TNF-α, IL-4, and IL-10) in serum. Data are represented as means ± SDs. * *p* < 0.05, ** *p* < 0.01, *** *p* < 0.001, **** *p* < 0.0001. ns, no significant difference. Each group had at least 6 biological replicates.

LM is commonly found in food processing environments and significantly threatens human health [41]. The following experiments were designed to investigate the potential of XJ-24 in preventing LM infection in mice, exploring the underlying physiological mechanisms. The weight change curves for mice in each group are shown in Figure 2B. From day 1 to day 15, the mice in the heat-inactivated XJ-24 (HK_XJ-24) group exhibited an increase in body weight over the feeding period, while those in the XJ-24 group showed no significant change compared to day 1. On day 15 post-infection with LM, a notable decrease in body weight ratio was seen in the LM, HK_XJ-24, and XJ-24 groups compared to the Control group (*p* < 0.05) (Figure 2E). However, compared to the LM group, XJ-24 pretreatment significantly alleviated LM-induced weight loss (*p* < 0.05), while HK_XJ-24 pretreatment showed minimal protective effects (*p* > 0.05). Furthermore, compared to the LM group, XJ-24 pretreatment notably decreased the bacterial load in both the spleen (*p* < 0.001) and colon (*p* < 0.05), whereas HK_XJ-24 pretreatment did not demonstrate similar protective effects (*p* > 0.05) (Figure 2C,D). Therefore, subsequent experiments will focus solely on active *P. acidilactici* XJ-24. These results suggest that XJ-24 can protect mice from LM infection and promote the host’s health.

### 2.6. Effects of XJ-24 Pretreatment on Serum Inflammatory Factors in Mice Infected with LM

Furthermore, pathogen invasion of the intestinal mucosa can increase intestinal permeability, allowing bacteria and their toxins to enter the bloodstream, activating innate and adaptive immune responses, and intensifying inflammation [42,43]. Therefore, we assessed the effect of LM infection on inflammatory response by measuring serum pro-inflammatory cytokine levels (Figure 2F–I). The LM challenge significantly elevated the serum concentrations of pro-inflammatory cytokines IL-6, TNF-α, IFN-γ (*p* < 0.0001), and IL-1β (*p* < 0.001) in mice compared to the Control group. Nevertheless, pretreatment with XJ-24 significantly reduced this trend, with serum pro-inflammatory cytokine levels showing no significant difference compared to the Control group (*p* > 0.05).

### 2.7. Effect of XJ-24 Pretreatment on Tissue Damage and Intestinal Barrier Integrity in Mice Infected with LM

LM exhibits strong invasiveness and virulence, capable of disrupting tight junction proteins by secreting pathogenic factors, thus exacerbating the inflammatory response and leading to severe intestinal infections [3]. Consequently, restoring tight junction proteins can be considered an effective target for improving intestinal barrier dysfunction [2]. In this study, we used C57BL/6J mice, which do not express human E-cadherin (hE-cadherin), a key receptor for the LM surface protein Internalin A (InlA) that facilitates bacterial invasion of intestinal epithelial cells [44]. Since murine E-cadherin (mE-cadherin) has a lower affinity for InlA, the LM infection model in C57BL/6J mice may differ from that in humans [45]. Previous studies have shown that in mouse models lacking hE-cadherin, LM primarily invades through E-cadherin-independent pathways, such as uptake by antigen-presenting cells or disruption of the intestinal barrier [46,47]. Therefore, our study focused on the role of XJ-24 in enhancing host immune defense and maintaining intestinal barrier integrity rather than investigating the E-cadherin-mediated LM invasion mechanism.

**Figure 3:**
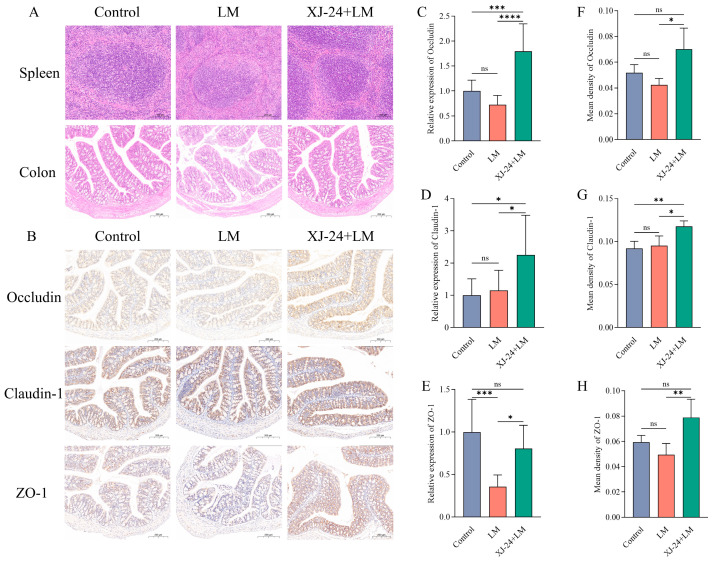
Effect of XJ-24 on the histological structure and intestinal barrier integrity in the LM infected mice. (**A**) Histomorphometric analysis of colon and spleen by H&E staining. The arrows indicate the damaged spots, (**B**,**F**–**H**) immunohistochemical analysis of Occludin, Claudin-1, and ZO-1, (**C**–**E**) the mRNA expression levels of Occludin, Claudin-1, and ZO-1 in the colon tissues. Data are represented as means ± SDs. * *p* < 0.05, ** *p* < 0.01, *** *p* < 0.001, **** *p* < 0.0001. ns, no significant difference. Each group had at least 6 biological replicates.

Histopathological analysis of H&E-stained sections of the colon and spleen revealed significant epithelial villus damage and severe mucosal edema in the colon in the LM group, indicating substantial tissue disruption (Figure 3A). Notably, pretreatment with XJ-24 significantly mitigated these pathological alterations. Additionally, histopathological changes were observed in the spleens of the LM group, including lymphocyte depletion and irregular white pulp morphology. However, pretreatment with XJ-24 effectively reduced these abnormalities (Figure 3A).

According to immunohistochemical analysis, the staining intensities of Occludin, Claudin-1, and ZO-1 did not significantly change between the LM and Control groups. (*p* > 0.05) (Figure 3B,F–H). However, the staining intensities of these three epithelial tight junction proteins were notably increased in the XJ-24 group relative to the LM group. (*p* < 0.05). Additionally, qPCR results revealed that the mRNA expression levels of Occludin, Claudin-1, and ZO-1 in colonic tissue were significantly elevated in the XJ-24 group compared to those in the LM group (*p* < 0.05), consistent with the immunohistochemical findings (Figure 3C,D). These results suggest that XJ-24 pretreatment can enhance intestinal barrier function and maintain intestinal health, thereby counteracting inflammation and intestinal barrier disruption induced by LM.

Although the absence of hE-cadherin in our model may limit the direct extrapolation of our findings to human LM infections, our results strongly support the potential of XJ-24 in preventing LM-induced intestinal damage.

### 2.8. Effect of XJ-24 Pretreatment on the Gut Microbiota Structure

The human gut microbiota is an extensive microbial ecosystem for various physiological processes and the maintenance of overall health [48]. Furthermore, microbiota-mediated colonization resistance serves as a vital defense mechanism against numerous intestinal pathogens, including LM [49].

**Figure 4:**
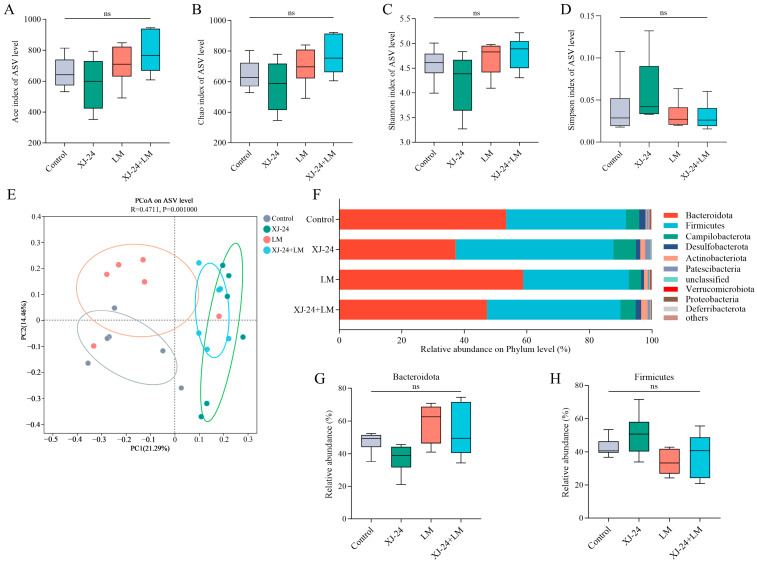
Mice fecal microbiota diversity. (**A**) Ace index, (**B**) Chao index, (**C**) Shannon index, (**D**) Simpson index, (**E**) principal coordinates analysis (PCoA). XJ-24 induces distinct bacterial composition in mouse feces. (**F**) Relative abundance of the intestinal microbiome in each group at the phylum level, (**G**) relative abundance of Bacteroidetes, (**H**) relative abundance of Firmicutes. Data are represented as means ± SDs. ns, no significant difference. Each group had at least 6 biological replicates.

The impact of XJ-24 pretreatment on the intestinal microbiota in mice was evaluated by taxonomic analysis using 16S rDNA gene sequencing. The findings revealed that XJ-24 pretreatment had no significant effect on the α-diversity of the gut microbiota. (*p* > 0.05) (Figure 4A–D). However, principal coordinate analysis (PCoA) demonstrated distinct clustering patterns in the gut microbiota of mice treated with XJ-24 compared to the control and LM groups (Figure 4E). Following XJ-24 pretreatment, the x-axis distances of the XJ-24 and XJ-24+LM groups were more divergent from the control group than the LM group. These findings suggest that a 14-day pretreatment with XJ-24 induced changes in the gut microbiota composition of mice. Similarly, Qiao reported that a 14-day pretreatment with different *P. acidilactici* strains modulated gut microbiota composition, with slight decreases in microbial diversity and evenness [50]. Further taxonomic analysis, presented below, revealed specific alterations in microbial composition, including shifts in beneficial and potentially harmful bacteria.

Further analysis of 16S rDNA sequencing indicated structural alterations in the gut microbiota across the different mice groups. At the phylum level, the gut microbiota was predominantly composed of Bacteroidetes, Firmicutes, and Proteobacteria, together constituting over 90% of the total microbiota (Figure 4F). Following 14 days of XJ-24 pretreatment, the proportions of Firmicutes and Bacteroidetes in the gut microbiota remained stable (*p* > 0.05) (Figure 4G,H). After the LM challenge, the LM and XJ-24+LM groups exhibited an increase in Bacteroidetes and a decrease in Firmicutes relative to the control group. Nonetheless, these variations did not reach statistical significance (*p* > 0.05).

**Figure 5:**
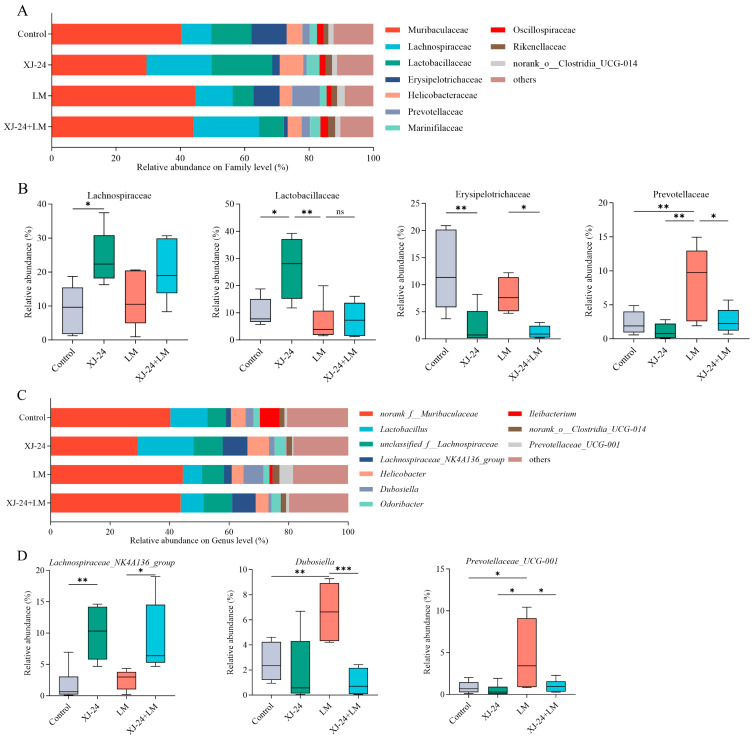
XJ-24 induces distinct bacterial composition in mouse feces. (**A**) Relative abundance of intestinal microbiome in each group at the family level, (**B**) difference in the relative abundance of specific intestinal bacteria at the family level between groups, (**C**) relative abundance of intestinal microbiome in each group at the genus level, (**D**) difference in the relative abundance of specific intestinal bacteria at the genus level between groups. Data are represented as means ± SDs. * *p* < 0.05, ** *p* < 0.01, *** *p* < 0.001. ns, no significant difference. Each group had at least 6 biological replicates.

At the family level, XJ-24 pretreatment significantly increased the relative abundances of *Lachnospiraceae* and *Lactobacillaceae* while reducing the relative abundance of *Erysipelotrichaceae* compared to the control group (*p* < 0.05) (Figure 5B). Post-LM challenge, the relative abundance of *Lactobacillaceae* in the XJ-24+LM group decreased significantly compared to pre-challenge levels (*p* < 0.05). Similarly, the LM challenge resulted in a marked rise in *Prevotellaceae* abundance in the LM group (*p* < 0.05), with no similar changes observed in the other groups.

At the genus level, the relative abundance of *Lachnospiraceae_NK4A136_group* in the XJ-24 group remained consistently high both before and after the LM challenge (*p* < 0.05) (Figure 5D). Moreover, the LM group showed a notable increase in the relative abundances of *Dubosiella* and *Prevotellaceae_UCG-001* (*p* < 0.05).

**Figure 6:**
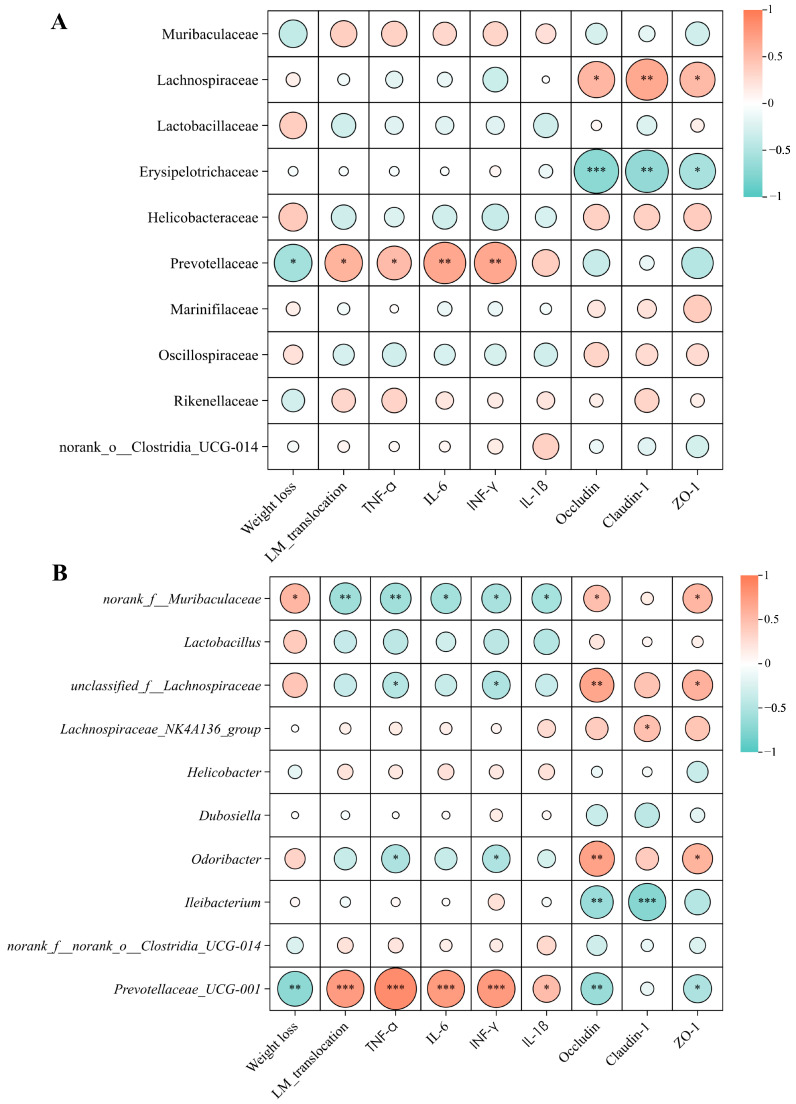
Correlation between microbial communities and phenotypic variables (weight loss, LM translocation in spleen, inflammatory cytokines, and the expression of tight junction proteins). (**A**) Correlation at the family level. (**B**) Correlation at the genus level. The size of the circles represents the absolute value of the correlation coefficient, with larger circles indicating stronger correlations. The heatmap shows the value of the correlation coefficient. * *p* < 0.05, ** *p* < 0.01, *** *p* < 0.001.

Previous studies have established a significant association between an increased relative abundance of *Prevotella* and various inflammatory conditions, including chronic intestinal inflammation, mucosal dysfunction, systemic inflammation, and periodontitis [51,52,53,54]. Additionally, interactions between *Prevotellaceae* and inflammasomes have been shown to promote inflammatory responses [55]. Spearman correlation analysis also revealed significant negative correlations between *Prevotellaceae*/*Prevotellaceae UCG-001* and pro-inflammatory cytokine levels as well as tight junction proteins. This suggests their potential role in inducing intestinal inflammation and impairing the function of the intestinal barrier in mice (Figure 6A,B). Furthermore, *Erysipelotrichaceae* have been linked to metabolic disorders and gastrointestinal inflammatory diseases in the host; for instance, an increased presence of *Erysipelotrichaceae* in the gut has been associated with conditions such as obesity, inflammatory bowel disease (IBD), and colorectal cancer [56,57,58,59]. In contrast, *Lachnospiraceae* has been shown to counteract pathogenic infections via mechanisms such as bacteriocin secretion, harmful compound degradation, and lactic acid regulation, thereby promoting gut stability [60,61]. *Lachnospiraceae_NK4A136_group* is recognized for its capacity to improve intestinal barrier function and preserve gut homeostasis through the production of short-chain fatty acids, preventing pathogen colonization and invasion, and reducing mucosal inflammation [62]. *Lactobacillaceae*, a diverse family of lactic acid bacteria prevalent in human and animal gut microbiomes, is essential for immune regulation and protecting against pathogens [63]. A correlation analysis also demonstrated that *Lachnospiraceae*, including *unclassified_f__Lachnospiraceae* and *Lachnospiraceae_NK4A136_group* within this family, exhibited significant positive correlations with tight junction proteins, whereas *Erysipelotrichaceae* showed a significant negative correlation.

These results indicate that XJ-24 modulates gut microbiota composition in mice under a normal diet, promoting the growth of beneficial bacteria while suppressing harmful bacteria, thereby increasing resistance to LM infection. Nevertheless, additional investigations are required to validate whether alterations in the gut microbiota significantly contribute to XJ-24’s efficacy in preventing LM infection, potentially via fecal microbiota transplantation experiments.

## 3. Materials and Methods

### 3.1. Bacterial Strains and Growth Conditions

*P. acidilactici* XJ-24 (XJ-24), initially isolated from traditional Xinjiang yogurt curds, has been deposited in the China General Microbiological Culture Collection Center (CGMCC, No. 30078). *L. monocytogenes* ATCC^®^ 9115TM (LM) was stored at −80 °C in a Tryptic Soy Broth (TSB) medium supplemented with 25% glycerol at the laboratory. Both strains were subcultured twice at 37 °C for 24 h in Man–Rogosa–Sharpe agar (MRS) and TSB media, respectively (Baisi Biotechnology Co., Ltd., Hangzhou, China).

### 3.2. Whole Genomics Analysis of XJ-24

Whole-genome sequencing, assembly, and annotation of XJ-24 were conducted by Majorbio Bio-pharm Technology Co., Ltd. (Shanghai, China).

Genome sequencing and assembly. XJ-24 in the exponential growth phase was collected via centrifugation (4 °C, 8000× *g*, 10 min). Genomic DNA was extracted using a FastPure Stool DNA Isolation Kit (Majorbio Bio-pharm Technology Co., Ltd., Shanghai, China). The Illumina HiSeq (Illumina, San Diego, CA, USA) and PacBio Sequel platforms (Pacific Biosciences, Menlo Park, CA, USA) were used for whole-genome sequencing. Genome assembly was conducted using the short-read assembler SOAPdenovo2 2.04 (https://github.com/aquaskyline/SOAPdenovo2, accessed on 25 May 2024), optimizing next-generation sequencing data with various K-mer parameters to achieve high-quality contigs. Reads were aligned to the contigs, followed by local assembly and optimization based on paired-end and overlapping relationships, producing scaffolds. The complete genome was further assembled using Unicycler v0.4.8 (https://github.com/rrwick/Unicycler/releases, accessed on 25 May 2024), integrating third-generation sequencing data. Assembly corrections were performed using Pilon v1.22 (https://github.com/broadinstitute/pilon/releases/tag/v1.22, accessed on 25 May 2024), where overlapping regions exceeding a specified length at both ends of the final assembly were resolved through sequence circularization and trimming, resulting in complete chromosomal and plasmid sequences.

Coding sequence (CDS) prediction was performed using Glimmer 3.02 (http://ccb.jhu.edu/software/glimmer/index.shtml, accessed on 25 May 2024), GeneMarkS 4.3 (http://exon.gatech.edu/GeneMark/, accessed on 25 May 2024), and Prodigal 2.6.3 (https://github.com/hyattpd/Prodigal, accessed on 25 May 2024). tRNAs were predicted using tRNAscan-SE 2.0.12 (http://trna.ucsc.edu/software/, accessed on 25 May 2024), and rRNAs were identified with Barrnap 0.9 (https://github.com/tseemann/barrnap, accessed on 25 May 2024). Functional annotation of XJ-24 was conducted using various databases, including Gene Ontology (GO), Kyoto Encyclopedia of Genes and Genomes (KEGG), Clusters of Orthologous Groups of proteins (COG), the Virulence Factor Database (VFDB), and the Comprehensive Antibiotic Resistance Database (CARD).

### 3.3. Animal Study

#### 3.3.1. Preparation of Strains

The preparation method for the active strain XJ-24 involved inoculating activated XJ-24 into a fresh MRS medium at a 2% (*v*/*v*) volume ratio. The culture was incubated at 37 °C for 24 h and then centrifuged (4 °C, 8000× *g*, 10 min) to harvest the cells. The bacterial pellet was rinsed twice with phosphate-buffered saline (PBS, pH 7.4), resuspended, and adjusted to a final concentration of 1 × 10^9^ colony-forming units (CFU)/mL. The heat-inactivated strain (HK_XJ-24) was prepared by heating the XJ-24 suspension (adjusted to 1 × 10^9^ CFU/mL) at 90 °C for 30 min to ensure complete inactivation. The LM was activated by inoculating into fresh TSB medium at a 2% (*v*/*v*) ratio and incubating at 37 °C for 24 h. The culture was centrifuged (4 °C, 8000× *g*, 10 min) to extract the bacterial cells following incubation. After two PBS washes (pH 7.4), the pellet was resuspended and adjusted to a final concentration of 5 × 10^9^ CFU/mL.

#### 3.3.2. Animal Modeling and Grouping

The animal experiments were approved by the Animal Ethics Committee of Southwest University (Ethics Approval No. IACUC-20231030-01). Male C57BL/6J mice (specific pathogen-free, SPF grade) weighing 18–20 g and aged 5–6 weeks were kept in controlled environments with a temperature of 25 ± 1 °C and a relative humidity of 50 ± 10%. Following a week of acclimation, the mice were divided into four treatment groups at random, consisting of eight mice each: Control, LM, XJ-24, and HK_XJ-24. The experiment lasted 18 days (Figure 2A).

From days 1 to 14, 200 μL of sterile PBS (pH 7.4) was administered daily by oral gavage in both the Control and LM groups. Meanwhile, the XJ-24 and HK_XJ-24 groups were administered 200 μL of XJ-24 and HK_XJ-24, respectively. On day 15, the LM, XJ-24, and HK_XJ-24 groups were administered 200 μL of LM by oral gavage, while the Control group continued to receive 200 μL of PBS (pH 7.4). After a three-day observation period, the experiment ended on day 18.

#### 3.3.3. Preparation of Mouse Tissue Samples

On day 14, before the LM challenge, mouse feces were aseptically removed, flash-frozen in liquid nitrogen, and stored at −80 °C. On day 18, fecal samples were taken using the same aseptic technique. The mice underwent a 12-h fasting period, after which blood was collected via the ocular route, and the animals were euthanized by cervical dislocation. The blood samples were left to rest at room temperature for 2 h before centrifugation (4 °C, 4000× *g*, 10 min) to recover the serum. The colon and spleen were excised from the mice, and a portion was preserved for histological analysis and bacterial enumeration. The leftover colon tissue was promptly flash-frozen in liquid nitrogen and stored at −80 °C.

#### 3.3.4. Measurement of LM Colonization

The colonization of LM in the colon and spleen was assessed using PALCAM selective agar (Land Bridge Technology Co., Ltd., Beijing, China). Tissue samples were homogenized in sterile PBS (pH 7.4) and serially diluted in 10-fold steps. The diluted tissue homogenates were then spread onto PALCAM agar and incubated at 37 °C for 24 h to determine CFU. The number of CFUs per gram of tissue was used to compute the bacterial colony counts.

#### 3.3.5. Enzyme-Linked Immunosorbent Assay

The levels of pro-inflammatory cytokines, IL-1β, IL-6, TNF-α, and IFN-γ, in the serum of mice belonging to each group, were measured using ELISA kits (Ruixin Biotechnology Co., Ltd., Quanzhou, China).

#### 3.3.6. Histopathological Analysis and Immunohistochemistry

The histopathological analysis of colon and spleen tissues was assessed in slides stained with hematoxylin and eosin (H&E). The immunohistochemical analysis of colon tissue was performed to evaluate the expression levels of tight junction proteins, including Occludin, Claudin-1, and ZO-1. Servicebio Technology Co., Ltd. (Wuhan, China) performed all experiments in this section.

#### 3.3.7. Quantitative Real-Time PCR

Total RNA was extracted from colonic tissue using Trizol reagent (Accurate Biotechnology Co., Ltd., Hunan, China). A total of 1 μg RNA was reverse-transcribed into cDNA using RT SuperMix (Vazyme Biotech Co., Ltd., Nanjing, China). Quantitative real-time PCR (qPCR) was conducted on a CFX Connect system (Bio-Rad Laboratories, Hercules, CA, USA) using ArtiCan™ SYBR qPCR Mix (Tsingke Biotech Co., Ltd., Beijing, China) following the manufacturer’s protocol. Relative gene expression was determined using the 2^−ΔΔCt^ method, with GAPDH as the internal control. Primer sequences for the target genes, including mouse Occludin, Claudin-1, and ZO-1, were provided by Tsingke Biotech Co., Ltd. (Beijing, China) (Table S1).

#### 3.3.8. Sequencing and Analysis of Gut Microbiota

Six fecal samples from each group were randomly selected and sent to Majorbio Bio-Pharm Technology Co., Ltd. (Shanghai, China) for microbial diversity analysis. DNA was first extracted from the mouse feces samples using a Bacterial/fungal DNA extraction kit (magnetic beads) (Majorbio Bio-pharm Technology Co., Ltd., Shanghai, China). The concentration and purity of the extracted DNA were measured using a NanoDrop 2000 spectrophotometer (Thermo Fisher Scientific, Waltham, MA, USA), and its integrity was verified by 1% agarose gel electrophoresis. The V3–V4 hypervariable region of the 16S rRNA gene was amplified via PCR using primers 338F (5′-ACTCCTACGGGAGGCAGCAG-3′) and 806R (5′-GGACTACHVGGGTWTCTAAT-3′). PCR products were analyzed via 2% agarose gel electrophoresis, excised, and recovered using the AxyPrep DNA Gel Recovery Kit (Axygen Biotechnology, Union City, CA, USA). The purified PCR products were quantified using the QuantiFluor™-ST Blue Fluorescence Quantification System (Promega Corp., Madison, WI, USA). Sequencing was performed using the MiSeq PE 300/NovaSeq PE 250 platform (Illumina, San Diego, CA, USA). Following sequencing, paired-end (PE) reads were demultiplexed by sample. Quality control and filtering were applied to paired-end reads based on sequencing quality. The reads were subsequently merged based on overlap, producing high-quality data for downstream analyses. The high-quality data were processed using sequence denoising algorithms, such as DADA2 or Deblur, to generate Amplicon Sequence Variants (ASVs) that represent unique sequences and their relative abundance. Various statistical and visualization analyses were performed using ASV representative sequences and abundance data, including taxonomic classification, community diversity, and differential species analysis.

### 3.4. Statistical Analysis

The statistical analyses were conducted using GraphPad Prism 10 software (https://www.graphpad.com, accessed on 16 March 2025). Data from all groups were presented as mean ± SD. A one-way analysis of variance (ANOVA) was applied to assess differences between the mean values of the experimental groups. *p*-value < 0.05 was considered statistically significant.

## 4. Conclusions

In summary, whole-genome analysis indicates that XJ-24 demonstrates excellent safety and probiotic properties, highlighting its potential as a promising probiotic candidate. Animal studies demonstrated that XJ-24 pretreatment significantly reduced LM colonization, alleviated inflammatory responses, and preserved intestinal barrier integrity in mice. Furthermore, under a standard diet, XJ-24 pretreatment modulated gut microbiota composition in mice by promoting beneficial bacterial populations while reducing harmful ones. These findings provide compelling experimental evidence for the in vivo efficacy of XJ-24 in preventing LM infection, underscoring the potential of XJ-24-based strategies for infection control. However, due to significant metabolic differences between mice and humans, the efficacy of XJ-24 in humans warrants further validation. Future investigations will focus on evaluating the safety and effectiveness of XJ-24 in humans and elucidate its molecular mechanisms, thereby advancing its potential clinical applications.

## 5. Patents

A patent related to *P. acidilactici* XJ-24 described in this manuscript is pending approval. The application number is CN202410698005.X, filed on 31 May 2024.

## Figures and Tables

**Table 1:** Characteristics of the XJ-24 genome.

Attributes	Values
Genome Size (bp)	2,069,896
G + C Content (%)	42.18
Coding Gene Number	2010
Coding Gene Average Length (bp)	886.43
Genes Assigned to COGs	1620
rRNA	15
5S rRNA	5
16S rRNA	5
23S rRNA	5
tRNA	58
sRNA	27
Plasmids	1

**Table 2:** Probiotic characteristic-associated genes in XJ-24.

Gene Locus		Gene Name	Gene Function
Universal stress family protein	ACG4EF_00285	-	universal stress protein
	ACG4EF_01025	-	universal stress protein
	ACG4EF_01030	-	universal stress protein
	ACG4EF_01595	-	universal stress protein
	ACG4EF_03305	-	universal stress protein
	ACG4EF_07140	-	universal stress protein
Proteases and chaperones	ACG4EF_02365	*clpP*	ATP-dependent Clp endopeptidase proteolytic
	ACG4EF_05205	*hslU*	ATP-dependent protease ATPase subunit HslU
	ACG4EF_05210	*hslV*	ATP-dependent protease subunit HslV
	ACG4EF_05335	-	ATP-dependent Clp protease ATP-binding subunit
	ACG4EF_05700	*clpB*	ATP-dependent chaperone ClpB
	ACG4EF_06340	*clpX*	ATP-dependent Clp protease ATP-binding subunit ClpX
	ACG4EF_07860	-	ATP-dependent Clp protease ATP-binding subunit
Temperature stress	ACG4EF_02195	*groES*	co-chaperone GroES
	ACG4EF_02200	*groL*	chaperonin GroEL
	ACG4EF_04750	*hrcA*	heat-inducible transcriptional repressor HrcA
	ACG4EF_04760	*dnaK*	molecular chaperone DnaK
	ACG4EF_04765	*dnaJ*	molecular chaperone DnaJ
	ACG4EF_06525	*cspA*	cold-shock protein
	ACG4EF_07950	-	cold-shock protein
Bile tolerance	ACG4EF_01095	*arcD*	arginine-ornithine antiporter
	ACG4EF_01470	*nagB*	glucosamine-6-phosphate deaminase
	ACG4EF_08565	*pyrG*	CTP synthase
Acid tolerance	ACG4EF_07220	*atpC*	ATP synthase subunit epsilon
	ACG4EF_07225	*atpD*	ATP synthase subunit beta
	ACG4EF_07230	*atpG*	ATP synthase subunit gamma
	ACG4EF_07235	*atpA*	ATP synthase subunit alpha
	ACG4EF_07240	*atpH*	ATP synthase F1 subunit delta
	ACG4EF_07245	*atpF*	ATP synthase subunit B
	ACG4EF_07250	*atpE*	ATP synthase subunit C
	ACG4EF_07255	*atpB*	ATP synthase subunit A
	ACG4EF_06250	-	Na+/H+ antiporter NhaC family protein
Alkaline stress	ACG4EF_09110	*amaP*	alkaline shock response membrane anchor protein AmaP

## Data Availability

Data are contained within the article.

## Supplementary Materials

The following supporting information can be downloaded at https://www.mdpi.com/article/10.3390/antibiotics14030323/s1, Figure S1: A circular genomic map of the XJ-24 plasmid; Figure S2: COG database annotation of the XJ-24 whole genome; Figure S3: GO database annotation of the XJ-24 whole genome; Figure S4: KEGG database annotation of the XJ-24 whole genome; Figure S5: Gene clusters for bacteriocins were predicted using BAGEL4; Table S1: Quantitative PCR primers used in C57BL/6 mice; Table S2: Putative antibiotic resistance genes identified in the genome of XJ-24; Table S3: Putative virulence factors in the XJ-24.
