# Supplementary material for: Whole Genome Analysis of Pediococcus acidilactici XJ-24 and Its Role in Preventing Listeria monocytogenes ATCC® 19115TM Infection in C57BL/6 Mice
Source: Antibiotics (Basel). 2025 Mar 19;14(3):323. doi: 10.3390/antibiotics14030323 (PMC11939717; doi:10.3390/antibiotics14030323)
Supplement: Supplementary file 1 [file antibiotics-14-00323-s001.zip › antibiotics-3498989-supplementary.pdf]

Supporting Information for Publication

Whole Genome Analysis of *Pediococcus acidilactici* XJ-24 and Its Role in Preventing *Listeria monocytogenes* ATCC® 19115™ Infection in C57BL/6 Mice

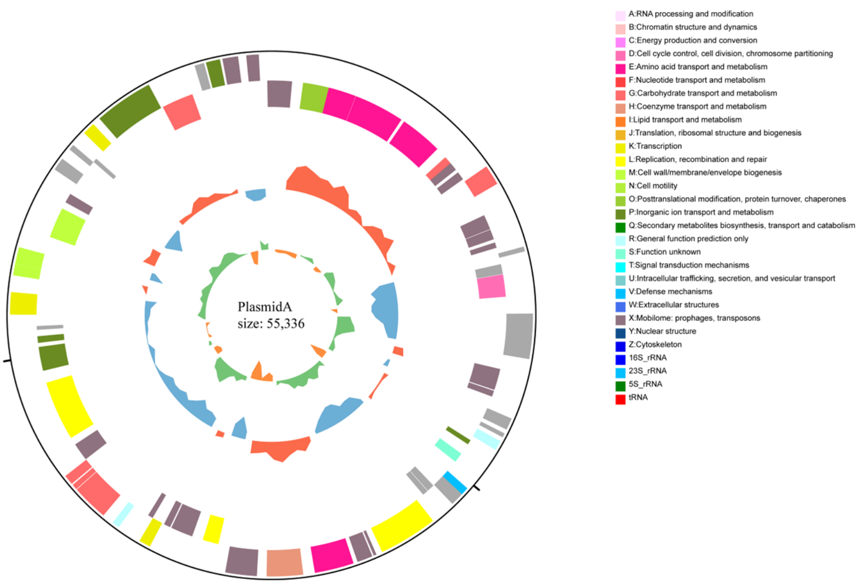

Figure S1. A circular genomic map of the XJ-24 plasmid.

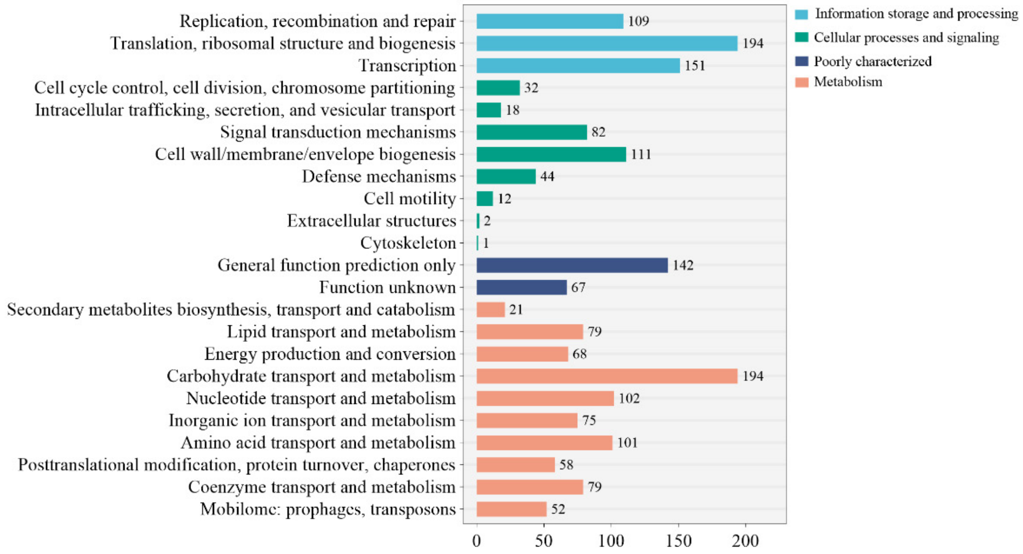

Figure S2. COG database annotation of the XJ-24 whole genome.

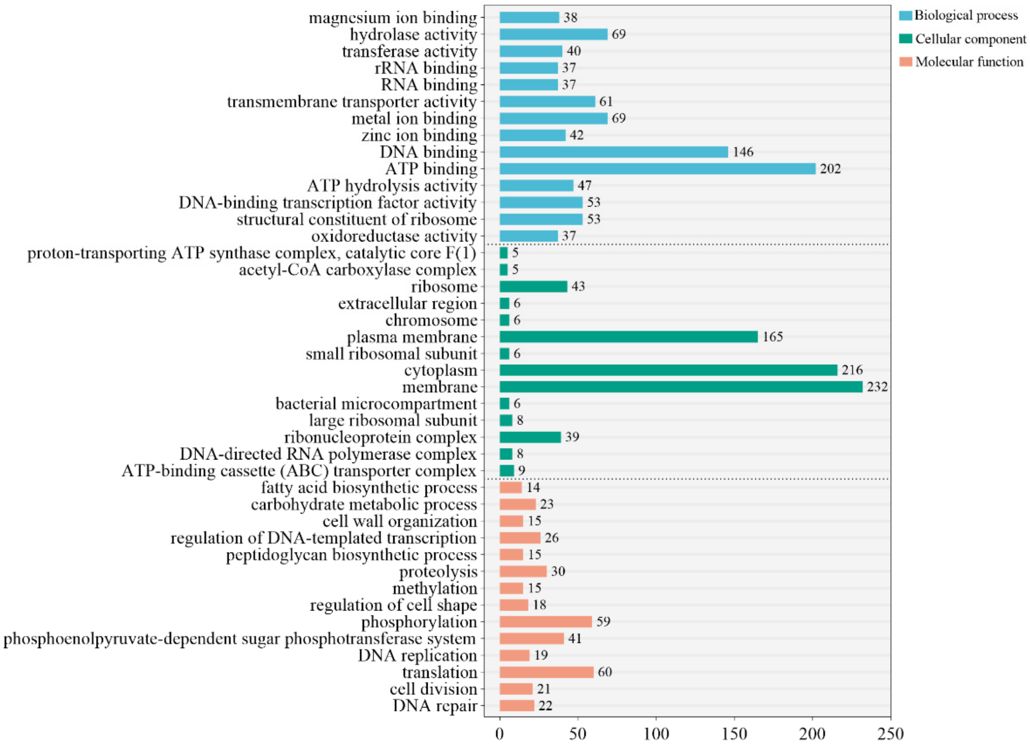

Figure S3. GO database annotation of the XJ-24 whole genome.

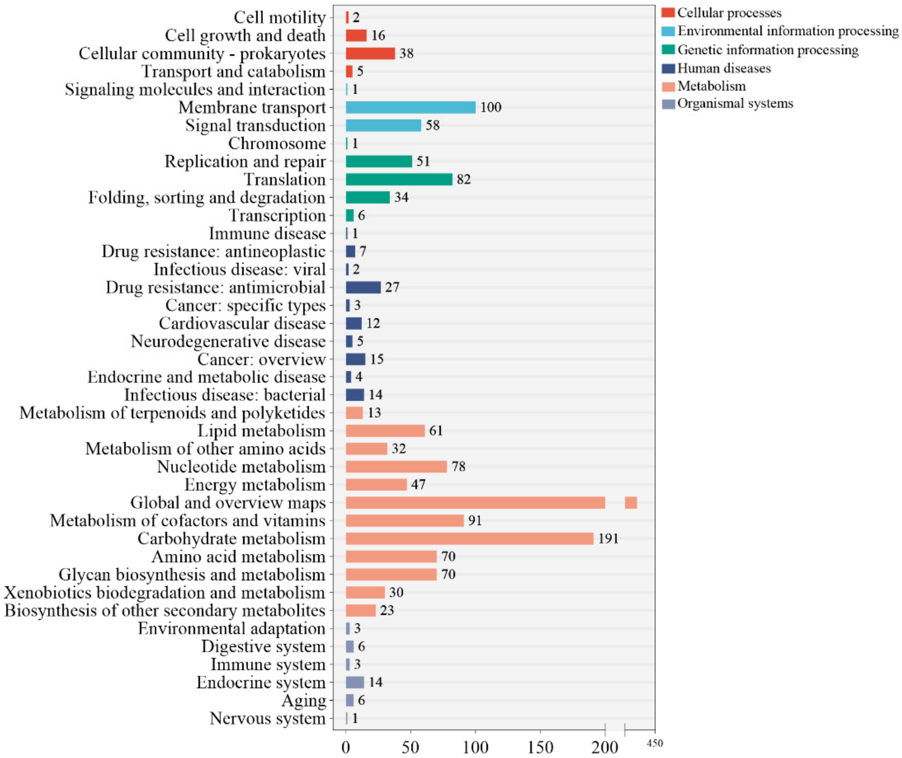

Figure S4. KEGG database annotation of the XJ-24 whole genome.

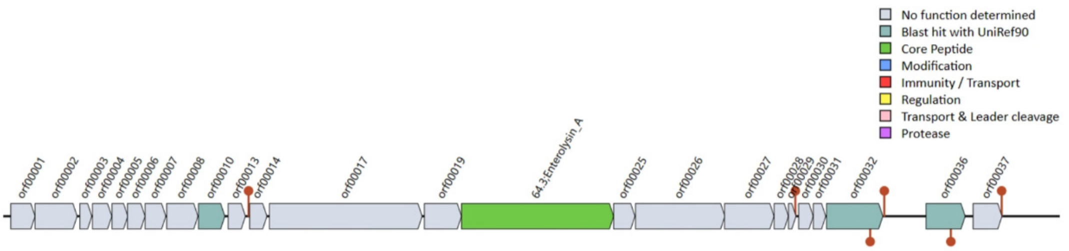

Figure S5. Gene clusters for bacteriocins were predicted using BAGEL4.

Table S1. Quantitative PCR primers used in C57BL/6 mice.

Genes	Forward primer (5′-3′)	Reverse primer (5′-3′)
GAPHD	ATTGTCAGCAATGCATCCTG	ATGGACTGTGGTCATGAGCC
Occludin	CAGCCTTCTGCTTCATCG	GTCGGGTTCACTCCCATTA
ZO-1	ACCCGAAACTGATGCTGTGGATAG	AAATGGCCGGGCAGAACTTGTGTA
Claudin-1	AGCACCGGGCAGATACAGT	GCCAATTACCATCAAGGCTCG

Table S2. Putative antibiotic resistance genes identified in the genome of XJ-24.

RGI Criteria	ARO Term	Detection Criteria	AMR Gene Family	Drug Class	Resistance Mechanism	Identity(%)
Strict	*sdrM*	protein homolog model	glycopeptide resistance gene cluster, vanT	glycopeptide antibiotic	antibiotic target alteration	31.69
Strict	*vanT gene in vanG cluster*	protein homolog model	major facilitator superfamily (MFS) antibiotic efflux pump	fluoroquinolone antibiotic, disinfecting agents and antiseptics	antibiotic efflux	34.81
Strict	*qacG*	protein homolog model	small multidrug resistance (SMR) antibiotic efflux pump	disinfecting agents and antiseptics	antibiotic efflux	48.11

Table S3. Putative virulence factors in the XJ-24.

Gene ID	Vfs	Identity(%)	COG Category
gene0480	ClpP	71.6	O
gene0469	Hyaluronic acid capsule	70.2	M
gene1285	EF-Tu	70.0	J
gene0837	Capsule	67.7	G
gene0447	GroL	65.7	O
gene0946	Capsule	64.0	I
gene0625	ClpE	60.8	O
gene0593	Capsule	60.3	M
gene1039	MsrAB	60.0	O
gene1585	ClpC	58.0	O
gene1957	Capsule	57.8	M
gene1154	SigA	56.3	K
gene0947	Capsule	51.1	I
gene1267	KatA	51.1	P

Only listed the annotated genes with more than 50% similarity.
